# A novel *MTTT* mutation m.15933G > A revealed in analysis of mitochondrial DNA in patients with suspected mitochondrial disease

**DOI:** 10.1186/s12881-017-0377-8

**Published:** 2017-02-10

**Authors:** Heidi K. Soini, Antti Väisänen, Mikko Kärppä, Reetta Hinttala, Laura Kytövuori, Jukka S. Moilanen, Johanna Uusimaa, Kari Majamaa

**Affiliations:** 1 Research Unit of Clinical Neuroscience, Neurology, University of Oulu, P.O. Box 5000, FI-90014 Oulu, Finland; 2 Medical Research Center Oulu, Oulu University Hospital and University of Oulu, P.O. Box 5000, FI-90014 Oulu, Finland; 3 Department of Neurology, Oulu University Hospital, P.O. Box 20, FI-90029 OYS Oulu, Finland; 4 PEDEGO Research Unit, Pediatrics, University of Oulu, P.O. Box 5000, FI-90014 Oulu, Finland; 5 Department of Pediatrics, Oulu University Hospital, P.O. Box 23, FI-90029 OYS Oulu, Finland; 6 Department of Clinical Genetics, Oulu University Hospital, P.O. Box 23, FI-90029 OYS Oulu, Finland; 7 PEDEGO Research Unit, Clinical Genetics, University of Oulu, P.O. Box 5000, FI-90014 Oulu, Finland

**Keywords:** mtDNA, Mitochondrial disease, *MTTT*, Insertion, Multiple deletions, *POLG1*

## Abstract

**Background:**

Mitochondrial diseases present with variable multi-organ symptoms. Common disease-causing mutations in mitochondrial DNA (mtDNA) are regularly screened in diagnostic work-up, but novel mutations may remain unnoticed.

**Methods:**

Patients (*N* = 66) with a clinical suspicion of mitochondrial disease were screened for their mtDNA coding region using conformation sensitive gel electrophoresis and sequencing. Long-PCR was used to detect deletions followed by *POLG1* sequencing in patients with multiple deletions.

**Results:**

We discovered three novel mtDNA variants that included m.8743G > C, m.11322A > G and m.15933G > A. The novel *MTTT* variant m.15933G > A is suggested to be pathogenic. Analysis revealed also multiple mtDNA deletions in two patients and five nonsynonymous variants that were putatively pathogenic according to *in-silico* prediction algorithms. In addition, a rare haplogroup H associated m.7585_7586insT variant was discovered.

**Conclusions:**

Among patients with a suspected mitochondrial disease, a novel *MTTT* variant m.15933G > A was discovered and is suggested to be pathogenic. In addition, several putatively pathogenic nonsynonymous variants and rare variants were found. These findings highlight the importance of coding region mtDNA screening among patients with clinical features suggesting a mitochondrial disease, but who lack the common mitochondrial disease mutations.

## Background

More than 200 pathogenic point mutations and large-scale rearrangements have been described in mitochondrial DNA (mtDNA) [1]. Some of the most common mitochondrial diseases caused by mutations in mtDNA are Kearns-Sayre syndrome, Leber’s hereditary optic neuropathy, myoclonic epilepsy with ragged red fibers syndrome, MELAS syndrome (mitochondrial encephalomyopathy, lactic acidosis with stroke-like episodes) and the syndrome of neuropathy, ataxia and retinitis pigmentosa (NARP). The prevalence of mitochondrial diseases caused by mtDNA mutations has been calculated to be 1:5000 in the adult population [1, 2] and the total prevalence rises up to 1:4300, when disorders caused by mutations in nuclear-encoded genes contributing to mitochondrial functions are included [3].

Screening of established pathogenic mtDNA and nDNA mutations is straightforward, but detection of novel or rare mtDNA mutations is more tedious. Such mutations may remain unnoticed, unless the entire mtDNA coding region is sequenced after excluding known mitochondrial disease mutations. However, conventional sequencing methods do not detect large mtDNA deletions, nor are they good in recognizing extremes of mtDNA heteroplasmy. It is also imperative to be able to analyse the pathogenic potential of novel or rare mtDNA mutations and to determine their frequency in the population [4], as mtDNA is highly variable by nature. There is also emerging evidence suggesting that polymorphic variation can influence disease phenotype and raise the risk for developing a mitochondrial disorder [5–9]. Furthermore, mtDNA variants have been suggested to increase the risk of common neurodegenerative diseases, such as Alzheimer’s and Parkinson’s disease [10–13]. In this sense, the Finnish population is ideal for detecting underlying genetic risk factors, as it is considered to be a genetically isolated, homogenous population.

In the present study, we screened the mtDNA coding region of 66 patients with a clinical suspicion of a mitochondrial disease. Our aim was to determine mtDNA sequence for rare mtDNA variants and to assess if they were pathogenic mutations or polymorphic risk factors. Conformation sensitive gel electrophoresis (CSGE) and sequencing were used to determine the mtDNA coding region of the patients and long-PCR was used to detect multiple mtDNA deletions. Polymerase γ gene (*POLG1*) was sequenced in multiple deletions patients.

## Methods

### Patients and population controls

Finnish patients (*N* = 66) with a clinically suspected mitochondrial disease were examined. Muscle samples (*N* = 21), blood samples (*N* = 36) or both (*N* = 9) were obtained. Common symptoms among selected patients included exercise intolerance, myopathy, encephalomyopathy, cardiomyopathy, diabetes mellitus, sensorineural hearing impairment and progressive external ophthalmoplegia. The patients consisted of sporadic cases or cases with similar symptoms in maternal relatives. The ethics committee of the University of Oulu has approved the study protocol and all patients gave a written informed consent for participation in the study.

Anonymous healthy blood donors (*N* = 480) were used as population controls. It was required that the donor and his or her mother were born in the same region, and that the donor did not report neurological ailments, diabetes or sensorineural hearing impairment in the family. MtDNA haplogroups have been determined in all of the 480 controls [14], the hypervariable segment I (HVSI) in the D-loop has been sequenced in 403 controls [15] and the entire mtDNA sequence has been determined in 192 controls [14]. The ethics committee of the Finnish Red Cross has approved the study protocol.

### Molecular methods

Total DNA was extracted from blood by using the QIAmp Blood Kit (Qiagen, Hilden, Germany). Total DNA from muscle samples was extracted by using Wizard® Genomic DNA purification kit (Promega, Madison, WI, U.S.A.) and standard tissue DNA extraction method using phenol and chloroform. MtDNA haplogroups were determined by restriction fragment analysis [16].

Forty-one samples were analysed by using conformation sensitive gel electrophoresis (CSGE) as previously described [17, 18]. The mtDNA coding region, nucleotides m.577-m.16090, was amplified in 63 partially overlapping fragments. Each amplified fragment was mixed with the corresponding fragment amplified on a control template with a known sequence. Heteroduplex formation was also allowed to occur autogenously enabling the detection of heteroplasmic mutations. Heteroduplexes that differed in mobility in CSGE were sequenced (ABI PRISM ™ 377 Sequencer using DYEnamic ET Terminator Cycle Sequencing Kit; Amersham Pharmacia Biotech Inc., Buckinghamshire, U.K.) after purification with *exonuclease* I and *shrimp alkaline phosphatase* [19]. The primers used for sequencing were the same as those used for the amplification of the 63 CSGE fragments. Twenty-five samples were sequenced directly for their entire mtDNA. The mtDNA was first amplified in 11 fragments followed by sequencing using primers that were used for the amplification of mtDNA fragments.

Muscle DNA samples (*N* = 30) were examined for multiple mtDNA deletions by using long-PCR (Phusion expand XL-PCR, Boehringer Mannheim, Mannheim, Germany) as described previously [20]. Certain *POLG1* mutations are fairly common in the Finnish population and, hence, the entire *POLG1* gene (NCBI Reference Sequence: NM_002693) was sequenced in the two samples that were found to harbour multiple mtDNA deletions. Next-generation sequencing would have provided an effective tool for mtDNA analysis and heteroplasmy detection but, unfortunately, this method was beyond our resources at the time of investigation.

### Analysis of mtDNA variants

Sequences were compared to the revised Cambridge reference sequence [GenBank: NC_012920] [21] and to mtDNA sequences available in the Mitomap database http://www.mitomap.org [22]. Variants were considered rare if they occurred ≤ 10 times in the Mitomap database among 32,059 mtDNA sequences. Novel substitutions were confirmed by RFLP or by sequencing in both directions at least twice from different PCR products. The possibility of m.15933G > A being an artefact caused by nuclear mitochondrial DNA was examined by amplifying total mtDNA of patient 41 with long-PCR (XL-PCR) Expand Long Template PCR system kit (Boehringer Mannheim) as described previously [20] and by subsequently sequencing the mutation site with a forward primer at m.15714 and a reverse primer at m.16090. Conservation of nucleotides among species was evaluated using Mamit tRNA: Compilation of Mammalian mitochondrial tRNA genes http://mamit-tRNA.u-strasbg.fr [23] in the case of tRNA genes and GiiB-JST mtSAP evaluation http://mtsnp.tmig.or.jp/cgi-bin/mtsnp/specAlign/ctrlSpecAlignE.cgi in the case of structural mtDNA genes [24]. The pathogenicity of m.15933G > A was assessed by means of a multifactorial probability-based classification method PON-mt-tRNA that predicts the pathogenicity of single nucleotide substitutions in human mitochondrial tRNAs [25].

### Analysis of nonsynonymous mtDNA mutations

Nonsynonymous mtDNA mutations were analysed for their pathogenic potential by using PolyPhen-2 version 2.2.2 [26] http://genetics.bwh.harvard.edu/pph2/, SIFT BLink [27] http://sift.jcvi.org/www/SIFT_BLink_submit.html, PMut [28, 29] http://mmb2.pcb.ub.es/PMut/ and SNAP [30] http://rostlab.org/services/snap/ algorithms. Pathogenicity classes were ascertained according to the guidelines suggested by Wallis et al. 2013 and Claustres et al. 2014 [31, 32].

PolyPhen-2 calculates a naïve Bayes posterior probability for each mutation to assess its damaging potential. Mutations are then classified as *benign* if the probability is less than 50%, *possibly damaging* if the probability is greater than 50% and *probably damaging* if the probability is greater than 90%.

SIFT BLink and PMut algorithms are based on sequence homology. These algorithms assume that functionally important protein sequences are conserved in evolution, whereas diverse positions are unimportant to protein function. SIFT scores for nonsynonymous mutations range between 0–1 and scores of ≤ 0.05 are considered to be deleterious. PMut also uses information on sequence conservation and neural networks to predict whether a variant is benign or pathogenic. PMut provides a reliability index ranging from 0 (most unreliable) to 9 (most reliable). SNAP predicts functional effect by using conservation and functional information of proteins, such as secondary structure and solvent accessibility. A variant is assigned either neutral or non-neutral. A reliability index (0 being the most reliable prediction and 7 being the most unreliable) and an accuracy percentage is also provided.

Exact test of population differentiation as implemented in Arlequin 3.5 was used to compare frequencies of mtDNA haplogroups between cases and controls [33]. Phylogenetic networks of mtDNA sequences were constructed on the basis of median algorithm [34].

## Results

### MtDNA sequence variation and novel mutations

**Fig. 1:**
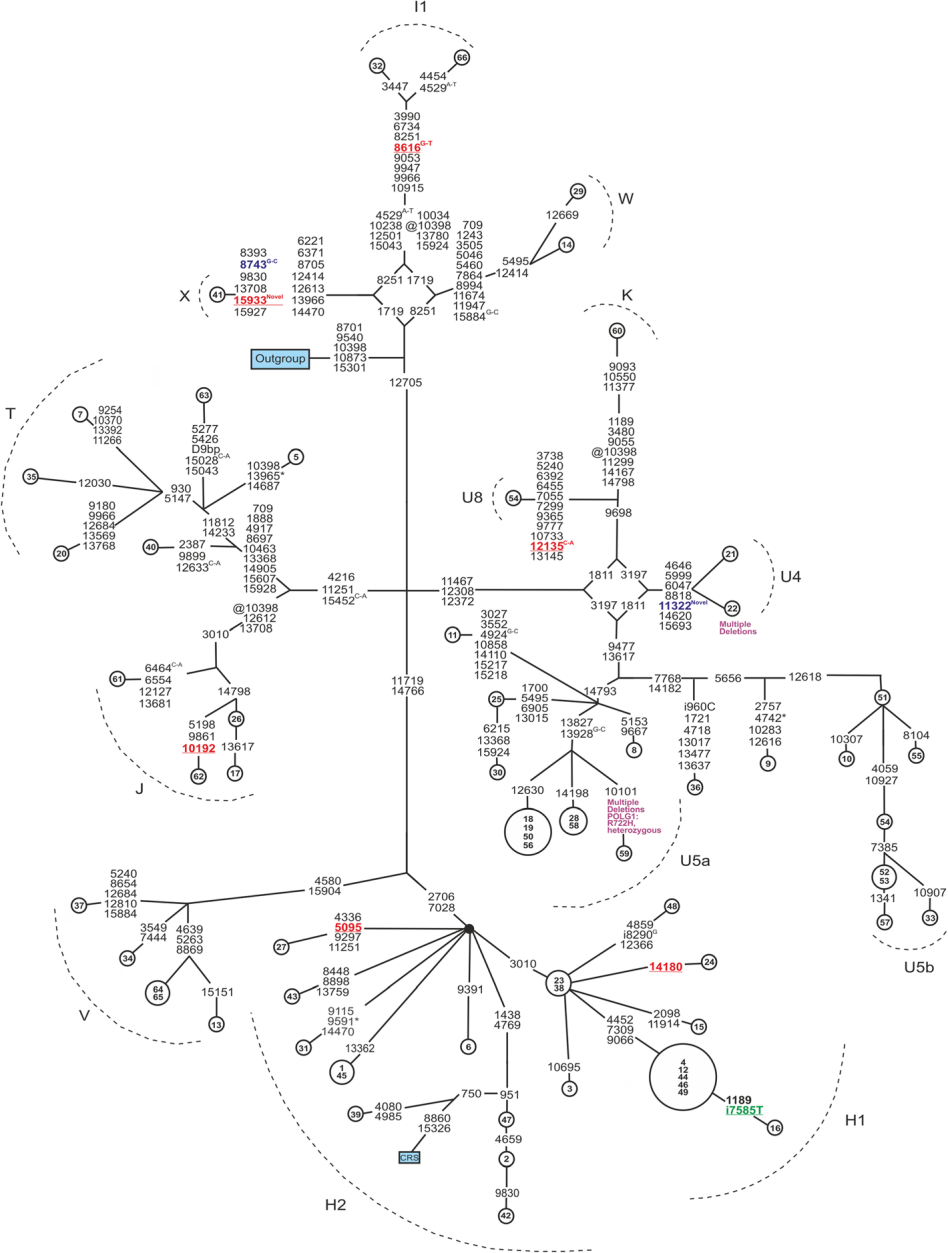
Phylogenetic network of the mtDNA coding region in 66 patients with suspected mitochondrial disease. Patients are identified by numbers inside the nodes. *Outgroup* = an African sequence (GenBank: AF346980); *CRS* = the revised Cambridge Reference Sequence (GenBank: NC_012920). Unless marked otherwise, mtDNA variants are transitions. Superscript text indicates transversions. Insertions are marked with superscript (i), back mutations (@) and heteroplasmic mutations are marked with an asterisk (*). Underlined red font, variants predicted to be likely pathogenic, blue font novel mutations, green font rare mutations, purple font multiple mtDNA deletions and *POLG1* mutations

**Fig. 2:**
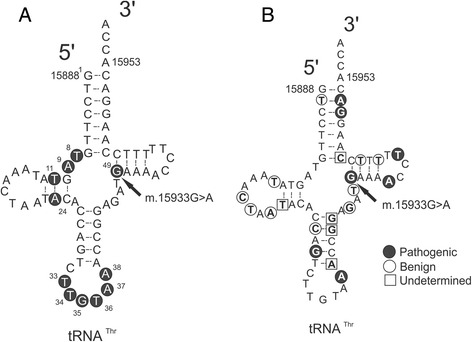
The m.15933G > A mutation in *MTTT*. **a** Invariable nucleotides among 135 mammal species in *MTTT* according to sequence alignment in Mamit tRNA [24]. **b** Human polymorphisms and pathogenic mutations in *MTTT*. Polymorphisms are marked with *white circles*, *black circles* denote confirmed pathogenic mutations and undetermined variants are marked with a *white square* ([24, 42])

The frequency of mtDNA haplogroups did not differ between the 66 patients with suspected mitochondrial disease and the controls. Three novel homoplasmic variants, m.8743G > C, m.11322A > G and m.15933G > A were discovered (Fig. 1). The m.11322A > G variant (p. N188S, *MTND4)* was found in two siblings, one (patient 21) with hypertrophic cardiomyopathy, chronic atrial fibrillation and sick sinus syndrome and the other (patient 22) with ptosis, restricted eye movements and exercise intolerance. The nucleotide position m.11322 is not notably conserved among 60 species which were investigated with GiiB-JST mtSAP evaluation http://mtsnp.tmig.or.jp/cgi-bin/mtsnp/specAlign/ctrlSpecAlignE.cgi [24]. The other two novel variants m.8743G > C (p.V73L, *MTATP6*) and m.15933G > A in *MTTT* were found in the muscle and blood of patient 41. The nucleotide at position m.8743 is not particularly conserved, whereas m.15933G is a highly conserved (Table 1, Fig. 2). The m.15933G > A mutation was predicted to be pathogenic with a probability score of 0.92 by PON-mt-tRNA method. Sequencing revealed that the m.15933G > A variant was present in an amplicon obtained in a long-range PCR amplification covering the whole mtDNA of patient 41.

**Table 1:** Evolutionary conservation analysis of the novel m.15933G > A mutation in *MTTT* among mammals

	T-stem	T-loop
Patient 41	^49^ **C**TTT ^65^ **A**AAA	ACCTT
*Homo sapiens*	^49^ **C**TTT ^65^ GAAA	ACCTT
*Pan paniscus*	^49^ **C**TTA ^65^ GAAA	ACTTT
*Pan trogdolytes*	^49^ **C**TTC ^65^ GAAA	ACTTT
*Gorilla gorilla*	^49^ **C**TTC ^65^ GAAG	ACCTC
*Hylobates lar*	^49^ **C**TTC ^65^ GAAA	TCTTC
*Rattus norwegicus*	^49^ **C**TTC ^65^ GAAG	AGTCT
*Mus musculus*	^49^ **C**TTC ^65^ GAAG	ATCTT
*Bos taurus*	^49^ **C**CTC ^65^ GGAG	AACAC

Mammalian *MTTT* sequences covering the T-stem base pairs and the T-loop. The nucleotide position m.15933 in consensus position 49 and its pair in position 65 are shown in the superscripts and the nucleotides are underlined

Patient 41 is a 51-year old woman. Her athletic achievements did not match those of the other girls at school. In adulthood she has had poor muscle fitness, and exercise-induced muscle weakness and myalgia. In addition, she has mild dysphagia and choking tendency. In her forties a nonsymmetrical ptosis developed and her right eye was operated at age 47 years. Psoriatic skin rash developed at age 49 years. Clinical neurological examination and blood lactate were normal. Muscle histology revealed ragged red fibers (RRF) and COX-negative fibers.

In addition, three other variants were considered rare occurring ≤10 times in the Mitomap database of 32,059 sequences. The rare insertion m.7585_7586insT (*N* = 4 in Mitomap database) between *MTTD* and *MTCO2* was found in patient 16. This insertion adds a thymidine between the last nucleotide of *MTTD* and the first nucleotide of *MTCO2* (Fig. 2). Cloning revealed that the insertion was present in all 100 clones investigated suggesting that the mutation in muscle mtDNA is homoplasmic with high probability. The other rare variants were m.4924G > C (p. S152T, *MTND2*) and m.7706G > A (p. A41T, *MTCO2*). The frequency of the rare nonsynonymous variants among the 66 patients did not differ from that in 89 haplogroup-matched controls drawn from the population [14].

### *In-silico* analysis of nonsynonymous variants

All the nonsynonymous mtDNA variants (*N* = 47) discovered in the 66 patients were analysed for their pathogenic potential using PolyPhen-2, SIFT BLink, PMut and SNAP algorithms. Five of the mutations were predicted to be deleterious by at least three of the algorithms (Table 2). The highest probability in favour of a pathogenic effect was obtained for m.8616G > T (p. L30F, *MTATP6*). With the exception of m.5095 T > C (p.I209T, *MTND2*) and m.14180 T > C (p. Y165C, *MTND6*), the three other variants predicted to be deleterious have previously been reported to be haplogroup-associated.

**Table 2:** Nonsynonymous mtDNA variants predicted to be likely pathogenic

Variant	Protein change	Gene	PolyPhen-2 (%)^a^	SIFT Blink^b^	SNAP^c^	Pmut^d^	Haplogroup association	Patient haplogroup
m.5095 T > C	p.I209T	*MTND2*	99.8	0	Pat/58%	Pat/7	Multiple	H2
m.8616G > T	p.L30F	*MTATP6*	99.9	0	Neutral/85%	Pat/7	I1, M74	I1
m.10192C > T	p.S45F	*MTND3*	0	0.02	Pat/58%	Pat/6	J1	J1
m.12135C > A	p.S459Y	*MTND4*	90.6	0	Pat/63%	Pat/9	U8, B4	U8
m.14180 T > C	p.Y165C	*MTND6*	99.7	0.02	Pat/63%	Pat/7	L2, T2, D4	H1

^a^PolyPhen-2 estimated probability of the variant being damaging: probabilities ~50% are classified as possibly damaging and probabilities >90% are classified as probably damaging

^b^SIFT score ranges from 0 to 1. The amino acid substitution is predicted damaging if the score is ≤ 0.05, and tolerated if the score is > 0.05

^c^SNAP assigns variants as neutral or non-neutral. An expected accuracy percent is given ranging from 50 to 100%, lower than 50% accurate predictions are not reported

^d^PMut predicts a variant either pathogenic or neutral. A reliability index is given, ranging from 0 (most unreliable) to 9 (most reliable)

The algorithms used for prediction included PolyPhen-2, SNAP, SIFT BLink and PMut. Variants were considered to be likely pathogenic if at least three algorithms predicted a damaging effect

### Multiple mtDNA deletions

Muscle samples from 30 patients were analysed for multiple mtDNA deletions. Patients 59 and 22 (Fig. 1) were found to harbour multiple deletions. Patient 59 suffered from bilateral progressive external ophthalmoplegia, diplopia, ptosis and mild exercise intolerance. His muscle histology revealed numerous RRFs and COX-negative muscle fibers. He harboured a heterozygous p.R722H allele in *POLG1*, but no potentially pathogenic mtDNA mutations.

Patient 22 harboured multiple mtDNA deletions and the novel m.11322A > G variant (p. N188S, *MTND4*), but not *POLG1* mutations. Her daughter had increased lactate in blood. No mtDNA deletions were detected in her brother (patient 21).

## Discussion

We discovered three previously unreported mtDNA variants, three rare variants and a total of 47 nonsynonymous variants among 66 patients with suspected mitochondrial disease. In addition, two patients harbored multiple mtDNA deletions. One of the novel variants was m.15933G > A in *MTTT* in a patient with ptosis, mild muscle weakness, myalgia, mild dysphagia, psoriasis and with RRF and COX-negative fibers in skeletal muscle. This variant is situated at the base of the T stem of tRNA^Thr^ in tRNA consensus position 49, which is highly conserved among mammals, including its pair nucleotide at position 65 [35]. Nuclear mitochondrial DNA as a source of m.15933G > A was excluded by whole mitochondrial genome amplification and subsequent sequencing. The insertion m.15933_15934insG in the same position has been reported in one subject (GenBank: JQ702629.1) [36]. This insertion is not considered to be deleterious, because it makes the preceding variable loop one base pair longer but it retains the conserved m.15933G. On the other hand, the m.15933G > A removes the highly conserved G in the beginning of the T stem and decreases the length of the T stem to four base pairs (Fig. 2). Another example of a similar pair of a pathogenic mutation and a polymorphic insertion is present in the T stem of *MTTL2*, where m.12311 T > C is pathogenic and m.12311_12312insA is polymorphic variant [35, 37, 38]. Altogether six confirmed pathogenic mutations have been reported in *MTTT* [22, 23]. The symptoms of the patient, the conserved nature of the nucleotide site and the predicted high probability in favour of pathogenicity suggest that m.15933G > A is pathogenic, but further functional studies will be needed to discern the biochemical consequences of the mutation.

The patient with the novel m.15933G > A harboured also the novel variant m.8743G > C (p.V73L, *MTATP6*). This position holds a known polymorphism of m.8743G > A (p.V73M, *MTATP6*), but the transversion m.8743G > C has not been previously reported in databases. This nucleotide position is variable and the amino acid at this position is not evolutionarily conserved. We consider this novel variant unlikely to be pathogenic.

The three rare variants included the insertion m.7585_7586insT that was discovered in a patient with a multi-organ phenotype. The m.7585_7586insT variant is interesting, as it adds a nucleotide in between the contiguous *MTTD* and *MTCO2* genes and may interfere with translation by one of three mechanisms. First, the *MTTD* transcript could be one nucleotide longer thus interrupting its proper function. Alternatively, the m.7585_7586insT insertion may affect the translation of *MTCO2*. The third possible mechanism entails that the RNAase cleavage site in between *MTTD* and *MTCO2* is disrupted. However, m.7585_7586insT has previously been discovered in four subjects belonging to haplotype H35, three of whom are Finnish. Our patient belonged to haplotype H1 suggesting that m.7585_7586insT has arisen at least twice in this population. Additional studies are needed to verify, if m.7585_7586insT indeed affects the function of mitochondria. Unfortunately, only a few blood samples were available from this family and no autopsy had been performed on the proband after his death.

The frequency of rare nonsynonymous variants among the 66 patients did not differ from that in 89 haplogroup-matched controls drawn from the population [14]. Five out of the 47 nonsynonymous variants were predicted to be pathogenic in effect. Four algorithms were used including SNAP, SIFT BLink, PolyPhen-2 and PMut. These algorithms have been reported to accurately predict 76% of non-neutral variants, and SNAP is among the most accurate methods for predicting damaging variants [39]. We assumed the best candidates for pathogenic mutations to be those that three out of four algorithms predicted to be pathogenic. Three out of five predicted pathogenic variants were haplogroup-associated and, indeed, we found these variants in the same haplogroups as previously reported, suggesting that they are haplogroup-associated polymorphisms. On the other hand, m.5095 T > C (p.I209T, *MTND2*) has previously been reported in multiple haplogroups (B, C, T, A, U5a, R) [23], whereas we discovered it in haplogroup H. This patient presented with proximal muscle weakness and atrophy, orbicularis oculi muscle weakness, myalgia and a large psoriasis-type rash. Inflammatory changes were detected in muscle histology. The true pathogenicity of m.5095 T > C remains, however, unknown despite the high pathogenicity prediction score, as this mutation has been discovered in healthy individuals. Multiple mtDNA deletions were discovered in two patients aged 33 and 42 years with suspected mitochondrial disease. Multiple mtDNA deletions do occur normally in the course of healthy aging, but up to 48% of younger mitochondrial disease patients with multiple deletions harbour *POLG1* mutations [40]. We found a heterozygous p.R722H allele in one patient, while the other patient with multiple deletions did not harbour any *POLG1* mutations. The clinical features of the patient with the heterozygous p.R722H allele included progressive external ophthalmoplegia, diplopia, ptosis, mild exercise intolerance, and numerous RRFs and COX negative fibers in skeletal muscle. The p.R722H *POLG1* allele has not been associated with disease in a heterozygous state, whereas in the homozygous state it leads to progressive external ophthalmoplegia, sensorineural hearing impairment, diabetes mellitus, dysphagia, a limb myopathy and dementia [41]. Patient 22 with multiple deletions, but without *POLG1* mutations, also harboured a novel nonsynonymous mtDNA mutation m.11322A > G (p. N188S, *MTND4*) that was predicted to be neutral.

## Conclusions

We determined the coding region mtDNA sequence of 66 Finnish patients with suspected mitochondrial disease. Among the 47 nonsynonymous variants, five were predicted to be deleterious in effect by using four algorithms in parallel, but three of them were considered haplogroup-associated polymorphisms. A likely pathogenic novel mutation m.15933G > A (*MTTT*) was discovered in patient with a suspected mitochondrial disease. Sequencing of the mtDNA coding region is recommended for patients presenting with symptoms of a mitochondrial disorder but lacking the common disease-causing mutations in mtDNA.

## Abbreviations

COX: Cytochrome oxidase
CRS: The Cambridge reference sequence
CSGE: Conformation sensitive gel electrophoresis
D-loop: Displacement loop, non-coding mtDNA segment
HVSI: Hypervariable segment I
MELAS: Mitochondrial encephalomyopathy, lactic acidosis with stroke-like episodes
MtDNA: Mitochondrial DNA
*MTTT*: Mitochondrial tRNA Threonine gene
NARP: Neuropathy, ataxia and retinitis pigmentosa
nDNA: Nuclear DNA
*POLG1*: Polymerase γ gene
RFLP: Restriction fragment length polymorphism
RRF: Ragged red fiber
